# The role of rapid syndromic diagnostic testing of gastrointestinal pathogens as a clinical decision support tool in a pediatric emergency department

**DOI:** 10.1186/s12941-023-00662-3

**Published:** 2024-01-05

**Authors:** Hyun Mi Kang, In Hyuk Yoo, Dae Chul Jeong

**Affiliations:** 1 Department of Pediatrics, Seoul St. Mary’s Hospital, College of Medicine, The Catholic University of Korea, Seoul, Republic of Korea; 2 College of Medicine, Vaccine Bio Research Institute, The Catholic University of Korea, Seoul, Korea

**Keywords:** Diarrhea, Emergency department, Syndromic multiplex diagnostic testing, Children, Stool

## Abstract

**Purpose:**

This study aimed to investigate the role of rapid syndromic diagnostic testing of gastrointestinal pathogens as a clinical decision support tool in a pediatric emergency department (ED) by comparing clinical decision and patient outcome parameters pre- and post-implementation.

**Methods:**

This was a big data analytical study of children < 18 years old without any underlying diseases, that visited the ED with acute moderate to severe diarrhea during a 34-month period from 2018 to 2022 using Seoul St. Mary’s hospital’s healthcare corporate data warehouse to retrieve demographic, clinical, and laboratory parameters. Outcome measures pre- and post-implementation of a rapid syndromic multiplex gastrointestinal panel (GI panel) were compared.

**Results:**

A total of 4,184 patients’ data were included in the analyses. Broad spectrum antibiotics were prescribed at a significantly lower rate to patients presenting with acute infectious diarrhea at discharge from the ED (9.9% vs 15.8%,* P* < 0.001) as well as upon admission (52.2% vs 66.0%, *P* < 0.001) during the post-implementation period compared to the pre-implementation period. Although the duration of ED stay was found to be significantly longer (6.5 vs 5.5 h, *P* < 0.0001), the rate of ED revisit due to persistent or aggravated symptoms was significantly lower (Δ in intercept, β = -0.027; SE = 0.013; *P* = 0.041), and the admission rate at follow up after being discharged from the ED shown to be significantly lower during the post-implementation period compared to the pre-implementation period (0.8% vs. 2.1%, *P* = 0.001, respectively). No significant difference in disease progression was observed (*P* = 1.000).

**Conclusion:**

Using the GI panel in the ED was shown to decrease broad spectrum antibiotic prescribing practices and reduce revisits or admission at follow up by aiding clinical decisions and improving patient outcome.

## Introduction

Acute diarrhea remains a common cause of morbidity and mortality in infants and children worldwide [1]. In developed countries, although mortality due to complications arising from acute diarrhea has become uncommon [2], However, it remains one of the most common causes and chief complaints of emergency department (ED) visits in children, and moderate to severe symptoms often lead to hospitalization. Therefore, the health care burden and costs caused by acute diarrhea remains high throughout the world [2–4].

Although the most common cause of acute diarrhea is by bacterial or viral infections, identifying the causative pathogen is difficult with current gold standard conventional diagnostic methods. Therefore, in majority of children that visit the ED with acute diarrhea, treatment decisions rely on presumptions of pathogens based on presenting clinical symptoms, physical examination, blood test results, and local seasonal epidemiology [5, 6], combined with the severity of presenting symptoms of the patient [6, 7]. In children with moderate to severe acute diarrhea, identifying causative organisms early on in the disease course may aid in making tailored decisions such as isolation and transmission prevention interventions, duration of hydration and conservative management, and administration of antimicrobial therapy [8–11].

Conventional methods for identifying the causative pathogens of acute infectious diarrhea include bacterial stool cultures, immunoassays for viruses, microscopy or enzyme immunoassays for parasites, in-house PCRs, and commercial syndromic multiplex PCRs [12, 13]. Major limitations of cultures and immunoassays are the low sensitivity or positive yield and the turnaround time [14]. Therefore, it is difficult to actively utilize these diagnostic methods in actual ED settings. However, rapid diagnostic tools in the ED may provide more evidence-based, precise and tailored therapeutic decisions.

Syndromic multiplex panels for detection and identification of causative gastrointestinal (GI) pathogens from the stools of patients with acute diarrhea have shown to be highly sensitive [15–18], and major advantages include the rapid turnaround time and ability to detect the presence of a variety pathogens [15]. Therefore, unlike conventional tests, these syndromic panels can potentially be advantageous in ED settings where rapid confirmation or exclusion of certain pathogens within hours can reduce diagnostic error and guide timely management of patients to improve the quality of ED care. Nevertheless, controversies remain on the clinical utility of syndromic panels and interpretation of the identified microorganisms from stools due to a lack of studies in the pediatric population as well as targeted groups such as those visiting the ED [9, 19]. Furthermore, overuse of syndromic panels can be misleading, especially when multiple pathogens are detected, eliciting unnecessary interventions that can potentially be harmful to the patient. Therefore, as diagnostic tests are advancing, the importance of diagnostic stewardship is becoming critical in ensuring that appropriate diagnostic tests are prescribed to patients at the right time for optimal clinical care meanwhile limiting overuse and misuse.

The aim of this study was to investigate the role of syndromic multiplex diagnostic testing of gastrointestinal pathogens in children that visit the pediatric ED for acute diarrhea by evaluating clinical decision parameters and patient outcome parameters pre- and post-implementation.

## Methods

### Study participants

This was a retrospective big data study of children < 18 years old without any underlying diseases, that visited the ED with symptoms of acute diarrhea during a 34-month period from 2018 to 2022, at a tertiary care, academic medical center in Seoul, South Korea. The first 17 months of the study period (From April 1, 2018 to September 30, 2019) was the period before the rapid syndromic multiplex GI pathogen panel (GI panel) was incorporated, the pre-implementation period, and the latter 17 months of the study period (From April 1, 2021 to August 31, 2022) was the post-implementation period. Between the pre-implementation period and the post-implementation period, there was an introduction period in which the GI panel test system was introduced to allow the medical staff to adapt. The GI panel that was incorporation in the ED was the BioFire ® FilmArray ® GI Panel (BioFire diagnostics, Salt Lake City, United States), which is able to detect the following 22 pathogens: *Campylobacter* (*C. jejuni/C. coli /C. upsaliensis*), *Clostridioides* (Clostridium) *difficile* (toxin A/B), *Plesiomonas shigelloides*, *Salmonella*, *Yersinia enterocolitica*, *Vibrio* (*V. parahaemolyticus/V. vulnificus/V. cholerae*), Enteroaggregative *E. coli* (EAEC), Enteropathogenic *E. coli* (EPEC), Enterotoxigenic *E. coli* (ETEC) *lt/st*, Shiga-like toxin-producing *E. coli* (STEC) stx1/stx2, *E. coli* O157, *Shigella*/Enteroinvasive *E. coli* (EIEC), *Cryptosporidium*, *Cyclospora cayetanensis*, *Entamoeba histolytica*, *Giardia lamblia*, Adenovirus F40/41, Astrovirus, Norovirus GI/GII, Rotavirus A, and Sapovirus (I, II, IV, and V).

The inclusion criteria were as follows: Patients that were 1) below 18 years of age, 2) visited the ED for acute diarrhea, 3) acute symptoms that began within 72 h, and had moderate to severe diarrhea, with moderate defined as 6–9 stools/day and severe defined as > 10 stools/day [20]. The exclusion criteria were as follows: 1) immunocompromised patients, 2) patients with chronic underlying medical conditions, 3) patients with inflammatory bowel disease or other chronic gastrointestinal disorders, and 4) considered mild acute infectious diarrhea, defined as mean stool frequency of 5 or less stools/day and mild degree of dehydration were excluded. Patients with mild acute infectious diarrhea were excluded from the study because majority were discharged from the ED without blood or stool tests.

### Study design

This was a big data analytical study using Seoul St. Mary’s hospital’s healthcare data warehouse which encompasses each patient’s clinical information, laboratory and image results, vital signs, and administrative information (ie. ED arrival, ED discharge time, ward transfer time, laboratory specimen submission times, laboratory results reporting times, etc.). Patients that fit the inclusion criteria were included as study participants and the following demographic and clinical parameters were extracted from the hospital’s corporate data warehouse: date of visit, age at visit, sex, ED arrival time and date, ED discharge time and date, any revisits within 7 days after ED discharge, time and date of ward transfer from the ED, discharge date from ward, date of outpatient clinic follow up after ED, admission from outpatient clinic after ED discharge, time and date of isolation, all types of antibiotics administered, GI panel results, laboratory findings, image findings (including abdominal x-rays, CT, ultrasound), blood culture and stool culture results, and date of pediatric intensive care unit transfer. All patient data was de-identified of all identifiers after retrieval.

After the GI panel was implemented in the ED, it was prescribed to patients with symptoms of acute diarrhea of moderate to severe symptoms, with clinically suspicious infectious etiology. Whether to prescribe the GI panel to the patient or not was the decision of the clinician treating the patient in the ED, however, was mainly decided considering the following: 1) need for hospitalization, 2) severity of symptoms, 3) subjective decision as to whether the patient will benefit from the test results, and 4) agreement of the guardians. The unformed stool specimens were collected by Copan flocked swab (FLOQSwab™, Copan, Murrieta, United States) and transported in 2 mL of Cary-Blair medium which was then immediately submitted to the hospital’s laboratory. The results were provided within 2–3 h of stool submission.

Ethics approval made by Seoul St. Mary’s hospital’s Data Review Board and Institutional Review Board (IRB no. KC22RISI0669) in accordance with the Declaration of Helsinki. This study was retrospective in design and data used for this study did not include any identifiers and thus informed consent was waived by the review board.

### Outcome measures and definitions

The primary outcome was assessing the following four clinical decision parameters. First, the clinician’s decision to prescribe antibiotics was assessed by observing changes in the proportion of patients prescribed broad spectrum antibiotics pre- and post-implementation of the GI panel, regardless of the pathogen identified. Second, knowing the etiologic pathogen of acute diarrhea can possibly reduce the number of additional imaging modalities such as abdominal ultrasounds or abdominal computer tomography (CT) prescribed in the ED. Therefore, the physician’s decision to undergo additional imaging modalities in the ED to rule out other focuses such as surgical abdomen or hidden infections was assessed by observing changes in the number of ultrasounds and CTs. Third, changes in infection prevention and control measures was analyzed by observing changes in 1) the number of consults made to infectious disease specialist for appropriate transmission prevention interventions, and 2) time to achieving appropriate isolation measures in patients.

The secondary outcome was assessing the following patient outcome parameters pre- and post-implementation of the GI panel: 1) changes in the duration of ED stay, 2) the number of ED revisit within 7 days for the same episode of acute diarrhea, 3) hospitalization rate from the outpatient clinic after discharge from the ED, and 4) changes in disease progression rate or patients that eventually were treated in the intensive care unit.

### Definitions

The outcome measures were compared between the two periods, pre- and post-implementation of the GI panel. The length of ED stay was determined by ED discharge time minus ED arrival time. The hospitalization rate was defined as the number of patients admitted from the ED divided by the number of total patients that visited the ED. The length of hospitalization was defined as the time and date of discharge from the ward minus the time and date of ward transfer from the ED. The percentage of antibiotic usage was determined as the percentage of patients that were prescribed any type of antibiotics (intravenous or oral) for acute diarrhea. Appropriate isolation measure was defined as patients that were intervened with the appropriate transmission prevention isolation measure depending on the identified pathogen. Time to appropriate isolation measures was defined as the time and date of execution of appropriate isolation measure minus the time and date of admission from the ED. Broad spectrum antibiotics was defined as antibiotics that kills or inhibits both gram positive and gram negative bacteria or a wide range of bacteria that causes diseases [21].

### Statistical analyses

*P*-values were calculated using chi-square test for categorical variables and t-test or wilcoxon rank sum test for continuous variables. Linear regression analyses were used to observe trends in outcomes after the implementation of the syndromic GI panel test in the ED. Interrupted time series regression analysis was used to observe the statistical significance of the immediate effects of incorporating the GI panel in the ED. In this study, the interrupted time series (ITS) model estimated the immediate change associated with the time point (change in level) and the change in slope from the baseline trend to the post-time point direction (change in trend). We used the Durbin-Watson test to detect autocorrelation. At the same time, we used SAS Proc AUTOREG with the BACKSTEP option to automatically select the correct order of the autoregressive model through backward elimination from an initial full model with order (k) = 12. Model fit was assessed using visual plots (autocorrelation function, partial autocorrelation function white noise probabilities, and autocorrelation functions). All analyses were conducted using SAS version 9.4 (SAS Institute, Cary, NC, USA) and R software version 4.1.2 (R Foundation for Statistical Computing, Vienna, Austria). All tests were two sided, and a *P* value < 0.05 was regarded as statistically significant.

## Results

### Characteristics of Study participants

**Fig. 1:**
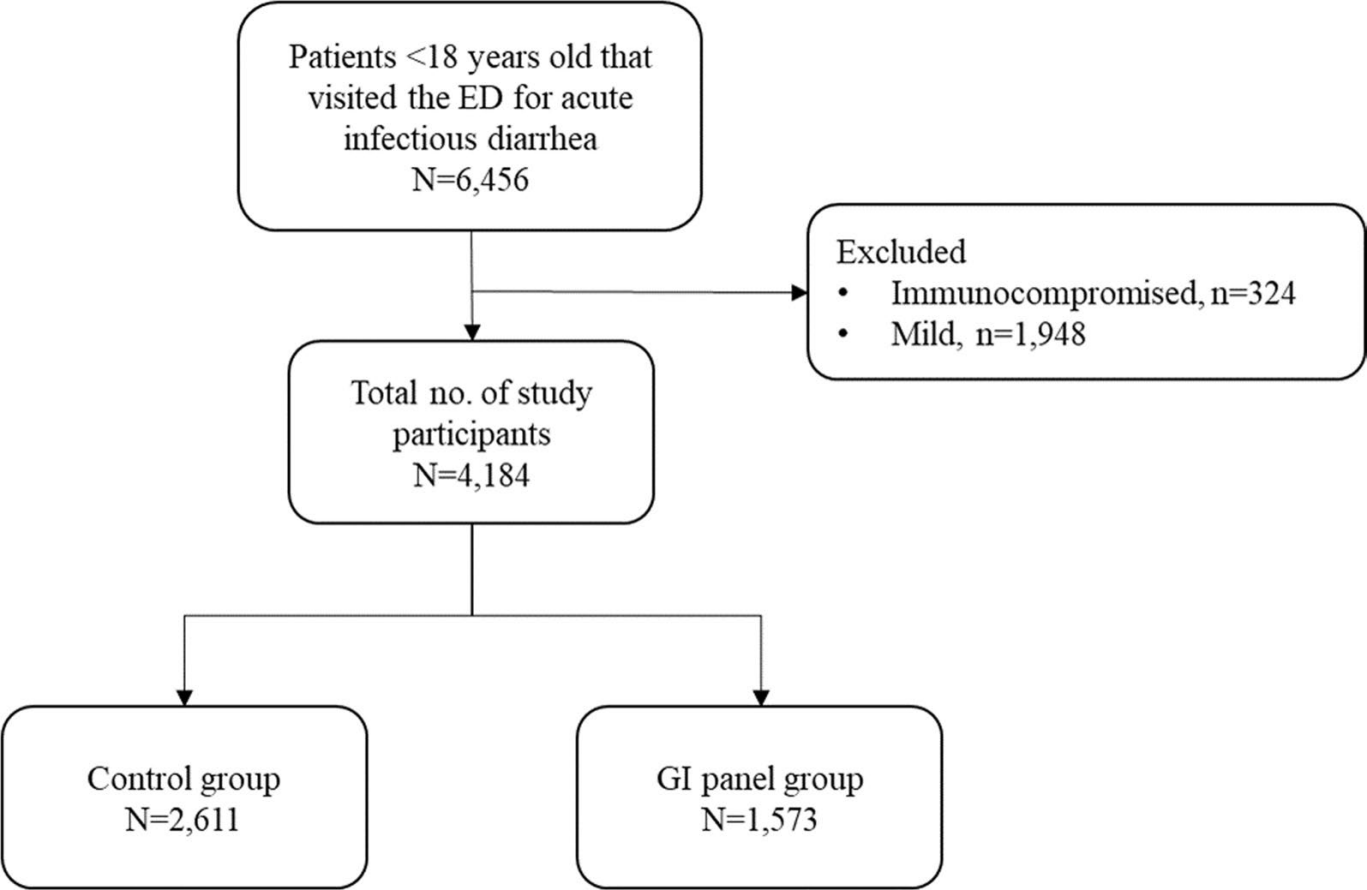
Flow chart of the patients included in this study. *ED* emergency department

**Fig. 2:**
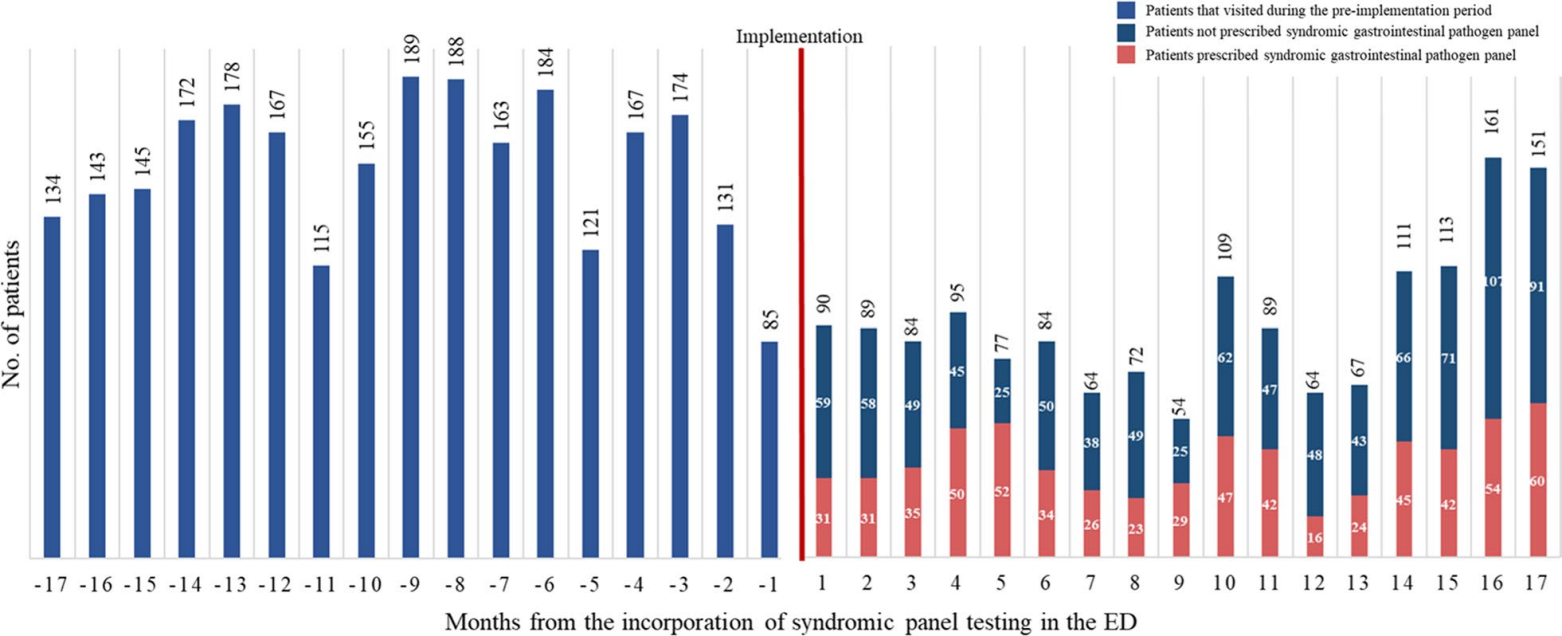
Monthly distribution of cases that visited the ED for symptoms of acute infectious diarrhea. Prior to the incorporation of the syndromic gastrointestinal panel test in the ED, an average 153.6 (standard deviation [SD] ± 28.2) patients visited the ED for acute infectious diarrhea, whereas after the incorporation, which was during the early COVID-19 period, an average 92.6 (SD ± 28.4) patients visited the ED. *COVID-19* coronavirus disease 2019, *ED* emergency department

**Fig. 3:**
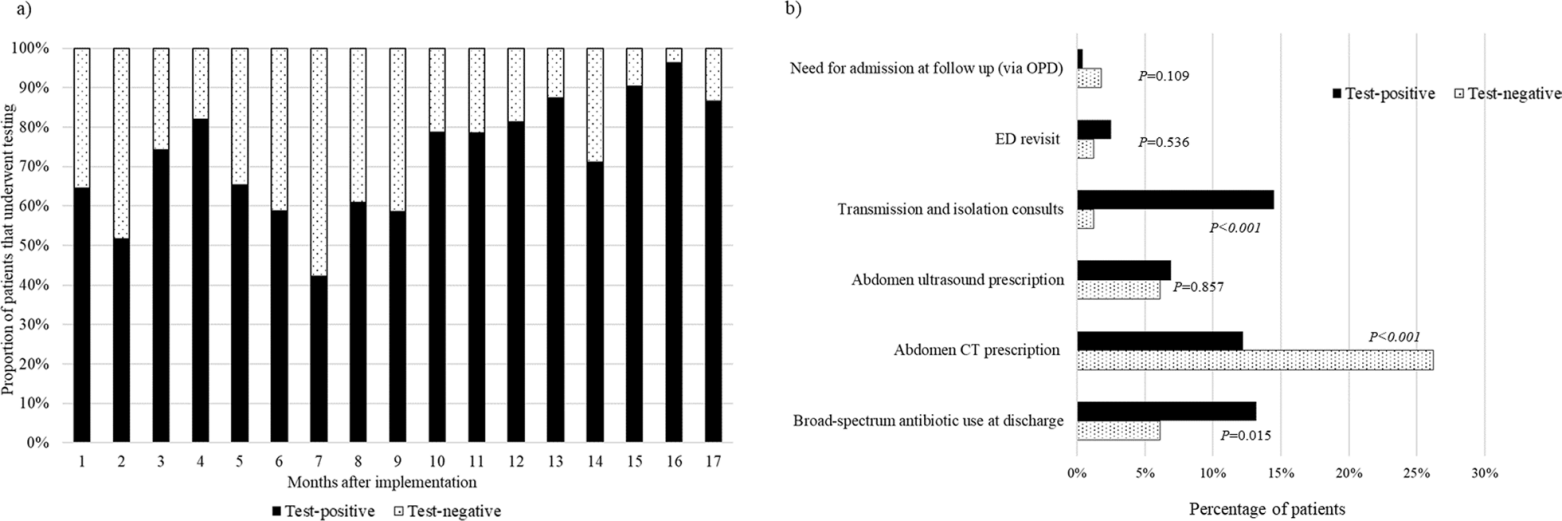
**a** Syndromic GI pathogen panel test positivity. An significant increase in test positivity rate was observed (β 2.5; 95% CI, 1.6–3.4; *P* < 0.001) as time passed after implementation, and **b** Subgroup analyses of outcome parameters in test-negative and test-positive patients. *CI *Confidence interval, *GI *Gastrointestinal

During the 34-month study period, a total of 4,184 children that visited the ED for acute infectious diarrhea that fit the inclusion criteria were included as study participants, and their demographic, clinical, and administrative data were retrieved. Of these, 62.4% (n = 2,611) visited the ED during the pre-implementation period, and 37.6% (n = 1,573) visited during the post-implementation period (Fig. 1). The monthly distribution and the proportion of patients are shown on Fig. 2. After the GI panel was implemented, as time passed, a significant increase in test positivity rate was observed (ꞵ 2.5; 95% CI, 1.6–3.4; *P* < 0.001) (Fig. 3a).

The mean age of the patients was 6.45 (standard deviation [SD] ± 5.19) years old in the pre-implementation and 7.10 (SD ± 5.08) years old in the post-implementation period (*P* < 0.001), with similar proportion of male patients (pre-, 52.8% vs. post-, 52.6%; *P* = 0.924) in both periods. In the post-implementation period, 40.8% (n = 641) had one or more pathogens identified by the syndromic GI panel. Initial laboratory findings of patients in both periods are found on Table 1. Other than elevated c-reactive protein and lactate dehydrogenase in both groups, all other parameters were in the normal range. Thus, although a significant difference in laboratory parameters were observed between the groups, the differences were considered to be clinically insignificant.

**Table 1:** Comparison of baseline characteristics of the patients during the pre-implementation and post-implementation period

	Pre-implementation n = 2,611	Post-implementation n = 1,573	*P*
Age, years			< 0.001
Mean ± SD	7.10 ± 5.08	6.45 ± 5.19	
Median (IQR)	6 (3–11)	5 (2–10)	
Sex, male			0.924
n (%)	1373 (52.6)	830 (52.8)	
Pathogen identified by PCR			–
n (%)	–	641 (40.8)	
Laboratory findings			
WBC, /mm^3^			0.0007
Mean ± SD	11.08 ± 4.85	10.53 ± 5.26	
Median(IQR)	10.11 (7.55, 13.74)	9.06 (6.76, 13.38)	
Hb, g/dL			< 0.0001
Mean ± SD	13.23 ± 1.19	12.94 ± 1.21	
Median(IQR)	13.20 (12.50, 13.90)	12.90 (12.20–13.60)	
PLT, /mm^3^			< 0.0001
Mean ± SD	290.35 ± 81.40	304.02 ± 86.29	
Median(IQR)	280 (238, 332)	292 (244, 351)	
CRP, mg/dL			0.6443
Mean ± SD	1.38 ± 2.79	1.34 ± 3.01	
Median(IQR)	0.20 (0.04, 1.37)	0.12 (0.03, 1.06)	
BUN, mg/dL			< 0.0001
Mean ± SD	13.66 ± 4.45	12.75 ± 4.58	
Median(IQR)	13.30 (10.40, 16.50)	12.20 (9.60, 15.40)	
Cr, mg/dL			< 0.0001
Mean ± SD	0.47 ± 0.18	0.43 ± 0.18	
Median(IQR)	0.43 (0.33, 0.57)	0.40 (0.31, 0.52)	
AST, mg/dL			0.0003
Mean ± SD	33.40 ± 32.53	30.62 ± 16.56	
Median(IQR)	30 (24, 38)	29 (21, 36)	
ALT, mg/dL			0.001
Mean ± SD	20.51 ± 40.86	17.62 ± 14.14	
Median(IQR)	15 (12, 20)	14 (11, 20)	
Total bilirubin, mg/dL			< 0.0001
Mean ± SD	0.57 ± 0.33	0.43 ± 0.31	
Median(IQR)	0.49 (0.35, 0.68)	0.35 (0.23, 0.51)	
Na, mg/dL			< 0.0001
Mean ± SD	139.80 ± 2.46	137.59 ± 2.54	
Median(IQR)	140 (138, 141)	138 (136, 139)	
K, mg/dL			0.4855
Mean ± SD	4.25 ± 0.42	4.24 ± 0.42	
Median(IQR)	4.2 (4.0, 4.5)	4.2 (4, 4.5)	
CL, mg/dL			< 0.0001
Mean ± SD	103.40 ± 2.82	102.75 ± 2.88	
Median(IQR)	104 (102, 105)	103 (101, 105)	
LDH, mg/dL			< 0.0001
Mean ± SD	568.08 ± 153.07	263.47 ± 70.48	
Median(IQR)	553 (471, 643)	258 (216, 298)	

### Primary outcomes

**Fig. 4:**
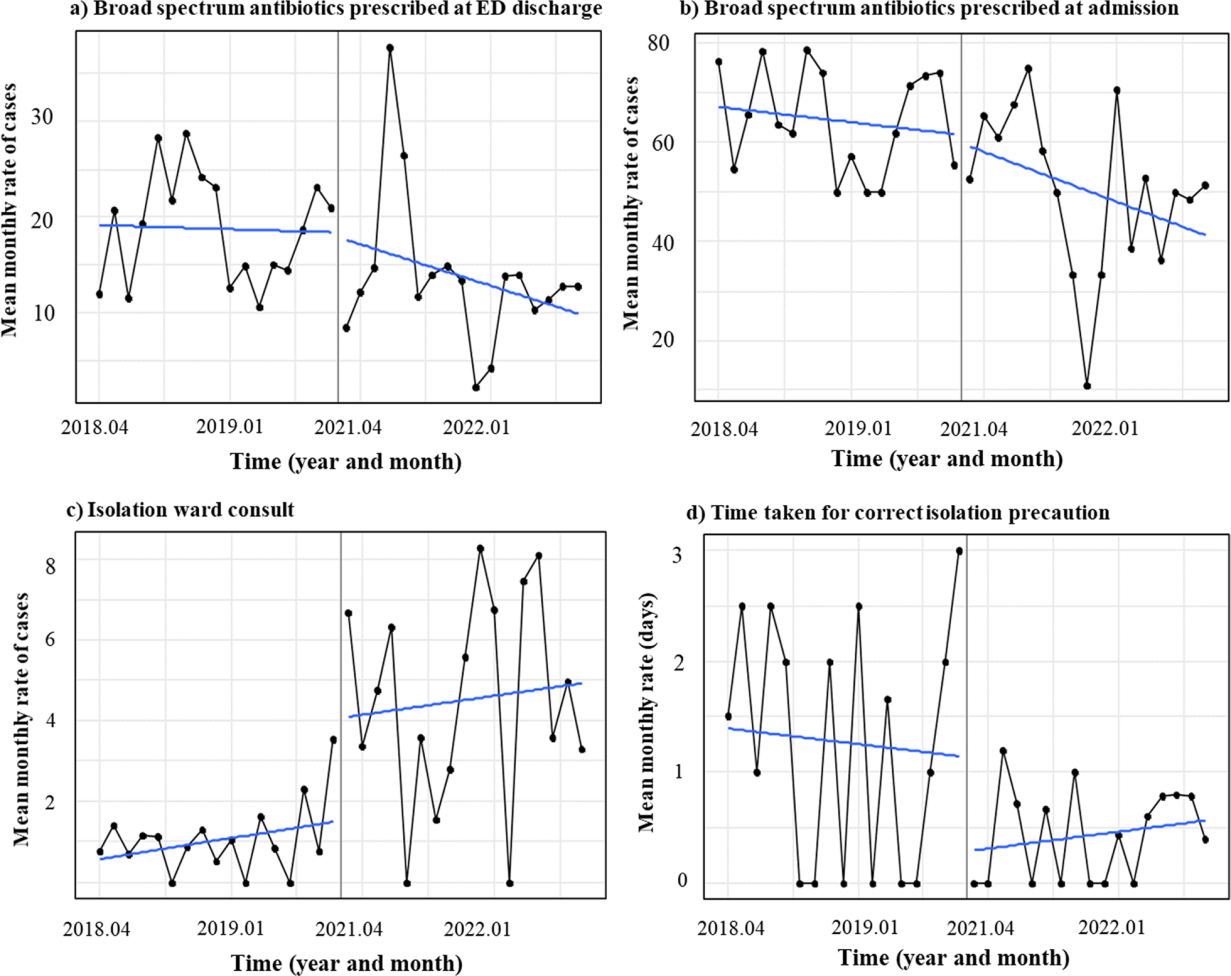
Primary outcome parameters. During the post-implementation period, a trend in decrease in no. of patients prescribed antibiotics **a** at ED discharge (ꞵ, − 0.745; 95% CI, -1.6–0.1; *P* = 0.093), and **b** a significant decrease at admission (ꞵ,− 1.2; 95% CI, − 2.0–− 0.4; *P* = 0.003). Furthermore, **c)** a significant immediate increase in the number of consults made to infectious disease specialists regarding isolation precautions (Δ in intercept, 0.031, SE, 0.010, *P* = 0.002) after the incorporation of syndromic gastrointestinal panel testing in the pediatric emergency department, as well as **d)** a significant immediate decrease in the time taken to exercise appropriate isolation precaution measures to patients at wards (Δ in intercept, -1.397, SE, 0.455, *P* = 0.003)

Overall, broad spectrum antibiotics were prescribed at a significantly lower rate to patients presenting with acute infectious diarrhea at discharge from the ED (9.9% vs 15.8%, *P* < 0.001) as well as upon admission (52.2% vs 66.0%,* P* < 0.001) during the post-implementation period compared to the pre-implementation period, respectively (Table 2). Furthermore, as time passed in the post-implementation period, a decreasing trend in number of patients prescribed broad spectrum antibiotics at ED discharge (ꞵ, -0.745; 95% CI, -1.6–0.1; *P* = 0.093), and a significant decrease at admission (ꞵ, -1.2; 95% CI, -2.0–-0.4; *P* = 0.003) was observed (Fig. 4a, b).

**Table 2:** Comparison of primary and secondary outcome measures before and after implementation of the syndromic gastrointestinal pathogen panel at the pediatric ED

	Mean monthly rate (%) (95% CI)		
	Pre-implementation	Post-implementation	P value
Primary outcome: Clinical decision parameters			
Broad-spectrum antibiotic use at discharge	15.8 (14.4–17.2)	9.9 (8.5–11.5)	< *0.001*
Broad-spectrum antibiotic use at admission	66.0 (61.1–70.6)	52.2 (47.1–57.3)	< *0.001*
Overall broad-spectrum antibiotic use	25.9 (24.2–27.6)	22.6 (20.5–24.7)	*0.016*
Abdomen CT prescription	15.0 (13.7–16.4)	17.2 (15.3–19.1)	*0.066*
Abdomen ultrasound prescription	5.4 (4.5–6.3)	4.4 (3.5–5.6)	*0.214*
Transmission and isolation consults	1.0 (0.7–1.5)	4.7 (3.7–5.9)	< *0.001*
Time taken for infection prevention intervention (days)	1.7 (1.7–1.8)	0.5 (0.5–0.6)	< *0.001*
Secondary outcomes: Patient outcome parameters			
Duration of ED stay (hours)	5.5 (5.4–5.7)	6.5 (6.3–6.8)	< *0.001*
ED revisit	5.2 (4.4–6.1)	2.6 (1.9–3.5)	< *0.001*
Need for admission at follow up (via OPD)	2.1 (1.6–2.7)	0.8 (0.4–1.4)	*0.030*
Disease progression rate	0.3 (0.2–0.7)	0.4 (0.1–0.8)	*1.000*

There were no significant differences in the amount of ultrasounds prescribed to patients during the pre- and post-implementation period (4.5% vs 5.4%, *P* = 0.214, respectively). A trend towards a higher number of abdominal CTs prescribed to patients in the post-implementation was observed, compared to the pre-implementation period (17.2% vs 15.0%, *P* = 0.066, respectively) (Table 2).

During the post-implementation period, the number of isolation ward consults made to ID specialists were significantly higher compared to the pre-implementation period (4.7% vs. 1.0%, *P* < 0.001) (Table 2). This was also shown in a time series analyses, where the number of consults regarding appropriate isolation methods to infectious disease specialists significantly and immediately increased post-implementation, in April, 2024 (Δ in intercept, β = 0.031; SE = 0.010; *P* = 0.002) (Fig. 4c). Furthermore, because in many hospitalized patients, the pathogen was identified in the ED prior to ward transfers, the time taken until appropriate isolation measure were executed in the wards after admission via ED were significantly lower (Δ in intercept, β = -1.400; SE = 0.455; *P* = 0.003) (Fig. 4d).

### Secondary outcomes

The duration of ED stay was found to be significantly longer (6.5 vs 5.5 h,* P* < 0.0001) in the post-implementation period compared to the pre-implementation period. However, the proportion of patients that re-visited the ED during the same episode of diarrhea decreased significantly (2.6 vs 5.2, *P* < 0.001) (Table 2). Furthermore, in a time series analyses, the rate of ED revisit due to persistent or aggravated symptoms was significantly lower during the post-implementation of GI panel in the ED (Δ in intercept, β = -0.027; SE = 0.013; *P* = 0.041). The admission rate from the outpatient department after being discharged early from the ED was shown to be significantly lower during the post-implementation period compared to the pre-implementation period (0.8% vs. 2.1%, *P* = 0.001, respectively) (Table 2). No significant difference in disease progression and need for admission to the intensive care unit was observed between the pre- and post-implementation period (0.4% vs 0.3%, *P* = 1.000, respectively).

### Subgroup analyses of GI panel test-positive and test-negative group

A subgroup analyses on patients that underwent GI panel testing during the post-implementation period was performed to observe how decision making changed according to the results of the study. We found that in test negative cases, the overall antibiotic usage was significantly lower in the test-negative group compared to the test positive group (6.1% vs. 13.2%, *P* = 0.009), and abdomen CTs were prescribed at a significantly higher percentage (26.2% vs. 12.2%, *P* < 0.001). In patients that were test-positive, transmission and isolation consults were prescribed at significantly higher percentages in the test-positive cases compared to test negative cases (14.5% vs. 1.2%, *P* < 0.001) (Fig. 3b).

### Factors associated with decisions to prescribe broad spectrum antibiotics in the ED

A multivariable analyses showed that the need for admission (*P* < 0.001), ID consults (*P* < 0.001), pathogen identified (*P* < 0.001), and patients that underwent abdomen CT (*P* < 0.001) were factors associated with the decision to prescribe broad spectrum antibiotics to patients in the ED with acute diarrhea (Table 3).

**Table 3:** Univariate and multivariate analyses of factors associated with decision to prescribe broad spectrum antibiotics for acute diarrhea in the ED

	Univariable				Multivariable			
	ß	OR (95% CI)	*SE*	*P*	ß	OR (95% CI)	*SE*	*P*
Control group	0.2	1.2 (1.0–1.4)	0.1	*0.016*				
Age	− 0.0	1.0 (1.0–1.0)	0.0	*0.337*				
Sex	− 0.1	0.9 (0.8–1.1)	0.1	*0.246*				
ED revisit	1.1	3.1 (2.3–4.2)	0.2	< *0.001*	0.5	1.6 (0.5–5.1)	0. 6	*0.418*
Admission via ED	2.0	7.3 (6.1–8.6)	0.1	< *0.001*	1.6	4.8 (3.3–7.1)	0.2	< *0.001*
Admission via opd	1.8	6.2 (3.7–10.3)	0.3	< *0.001*	3.3	26.1 (2.7–250.5)	1.2	*0.005*
ID consults for infection	0.9	2.5 (1.7–3.7)	0.2	< *0.001*	1.2	0.3 (0.2–0.6)	0.3	< *0.001*
GI panel test	0.7	2.1 (1.7–2.5)	0.1	< *0.001*				
Pathogen identified via GI PCR	0.5	1.7 (1.2–2.5)	0.2	*0.007*	1.0	2.6 (1.7–4.1)	0.2	< *0.001*
Abdomen CT	0.7	2.0 (1.7–2.4)	0.1	< *0.001*	1.0	2.8 (1.7–4.6)	0.2	< *0.001*
Ultrasound	0.7	2.0 (1.5–2.6)	0.1	< *0.001*	0.0	1.0 (0.5–2.0)	0.4	*0.962*
ICU admission	2.1	8.5 (2.7–26.7)	0.6	< *0.001*	1.7	5.4 (0.6–51.4)	1.2	*0.144*

## Discussion

Knowing the causative pathogens early on during the course of acute infectious diarrhea has several implications for treatment decisions and outcomes of patients. For example, if a virus is the cause in pediatric patients, whether or not dehydration occurs in the early stage and rehydration are most important [2, 22, 23]. If bacteria are the cause, it is necessary to decide whether to use antibiotics with consideration for the type of bacteria, severity of the disease, and risk factors of the patient [24–27]. Patients with confirmed norovirus acute gastroenteritis (AGE) are highly contagious compared to other pathogens, thus contact isolation is required if hospitalized, and isolation from kindergarten or school is required in young children during the symptomatic period even if not hospitalized [28, 29]. Pediatric patients with confirmed Shiga toxin-producing *E. coli* are at high risk of developing hemolytic uremic syndrome and may require hospitalization [30, 31]. As such, if causative pathogens can be identified early on during the course of disease, patients can receive a more specialized and tailored intervention.

The syndromic multiplex GI panel test is highly sensitive and enables rapid identification of the causative pathogens of infectious diarrhea [15–18]. Yet, cultures remain the gold standard for etiologic diagnoses of pathogens causing AGE and issues exist on the diagnostic value of detecting genetic material of pathogens because of the uncertainties pertaining to its viability and transmissibility [32, 33]. Therefore, evaluating its utility is extremely important in special settings, such as the ED.

Our study confirmed that certain benefits exist in the utilization of syndromic multiplex GI panels in the ED setting, and with correct diagnostic stewardship, targeted patient groups can receive tailored intervention with treatment decisions that can improve the quality of medical care. A major advantage of using the GI panel in the ED was the reduction in overall broad spectrum antibiotic prescriptions in the ED. Although majority of the etiologic pathogens causing AGE are viral, during the pre-implementation period, not knowing the pathogen led to more than 15% of the patients with moderate to severe diarrhea being prescribed broad spectrum antibiotics. Following the implementation of the GI panel which enabled early detection of pathogens, a significant decrease in the rate of antibiotic prescriptions was observed. A subgroup analyses of patients that underwent GI panel testing in the post-implementation period showed that a higher percentage of antibiotics were prescribed in the test-positive group and lower percentage in the test-negative group showing that antibiotic prescriptions could be more tailored according to the results of the GI panel.

Furthermore, a multivariable analyses showed that the decision to prescribe broad spectrum antibiotics were influenced by disease severity (need for admission), as well as objective parameters such as an identified pathogen, abdomen CT showing complications, or based on recommendations by ID specialists. A previous study also showed similar results in patients admitted for acute diarrhea, where one of the quality improvements in the management of children with moderate to severe acute diarrhea using the GI Panel was a significant reduction in antibiotic usage from 71.8% to 35.3% (*P* < 0.001) [8]. Unfortunately, although the GI panel includes major bacterial and viral causes of diarrhea, not all bacterial causes such as *Yersinia pseudotuberculosis* are included in the panel, therefore, more studies are needed before suggesting the possibility of the GI panel replacing stool cultures in certain patient groups.

Another important role of the syndromic multiplex GI panel in the ED was aiding in infection prevention measures. These results are presumed to be because early identification of pathogens that require isolation, such as norovirus and rotavirus, were mostly confirmed in the ED, leading to correct isolation interventions immediately upon admission which can ultimately decrease in-hospital transmissions and outbreaks. Because the GI panel can detect recent infections unrelated to the current symptoms, decisions based on the results of the GI panel need to be made with caution. In South Korea, hospitalized patients infected with highly contagious pathogens such as Norovirus and Rotavirus, as well as highly virulent pathogens such as Enterohemorrhagic *E. coli*, etc. are required standard and contact precaution plus potential single room isolation which is insured by the government. ID specialists are usually consulted to determine whether the detected organism is shedding from as past infection versus pathogen causing disease. As the number of collaborative consultations with ID specialists through GI panel testing increased consequently increasing related medical cost, in-hospital transmissions have simultaneously decreased, which have led to an overall benefit. In this group of patients, all patients with pathogens identified by the GI panel were symptomatic with diarrhea and therefore even though the pathogens detected may have been from a past recent infection, isolation precautions may have been beneficial in reducing transmission of other unidentified pathogens as well. Nevertheless, only relying on the results of the GI panel may lead to unnecessary isolation precautions which may be a burden in terms of limited isolation rooms and increase in medical costs.

Although the duration of ED stay was found to increase post-implementation of the syndromic panel, ED revisits decreased significantly as well. This shows that during the post-implementation period, by early detection of the pathogens, tailored and thorough interventions were made in the ED, which may have lengthened the duration of ED stay, however, improved patient outcome by reducing the number of patients that revisited the ED for the same symptoms. Although ED discharge may have been delayed due to the time taken to check results of the submitted GI panel test, which can be a disadvantage of the test, there was a significant decrease in the proportion of patients that were admission from the outpatient department at follow up after ED discharge as well, showing that the overall quality of ED care had improved.

When we first introduced the GI panel test to our hospital, it was expected that rapid identification of pathogens would lead to a decrease in the use of abdomen CT. However, after the introduction of the actual GI panel test, the number of abdomen CT scans actually increased, although the difference was not statistically significant. In a subgroup analyses of patients that underwent GI panel in the post-implementation period, we found that in test-negative patients, a significantly higher percentage of patients were prescribed abdominal CTs showing that knowing the pathogen in patients with moderate to severe symptoms can lead to a decrease in excessive and unnecessary examinations to rule out non-infectious etiologies that may require surgical interventions.

Our study has some limitations. The study period overlapped with the COVID-19 pandemic, so the number of patients during the post-implementation period was smaller compared to the pre-implementation period. This is a limitation of our study, as the reduced number of patients may affect the study results by affecting the patient population, clinical decision, and other results. Also, since this study was conducted as a retrospective chart review, there were inevitably limitations in the selection of subjects in the two groups and the analysis of the results. Additionally, patients that were discharged may have subsequently visited another hospital or healthcare system and be treated there subsequent to the initial measured encounter. These subsequent encounters that could not be identified may have affected the study’s outcomes. Finally, due to the nature of big data study, pathogen analyses could not be included. However, compared to other existing studies, the fact that the number of subjects is large is an advantage of our study that can reduce these limitations.

In conclusion, our study showed that in patients that visited the ED for moderate to severe diarrhea, the syndromic multiplex GI panel had an important role in reducing broad spectrum antibiotic use and increasing infection prevention measures. Although there was no clear benefit in reducing the duration of ED stay and disease progression, the overall quality of ED care was enhanced by reducing revisits or admission at follow up during the same episode of acute diarrhea. Further prospective studies are needed to understand the complete benefits of diagnostic stewardship of the syndromic multiplex GI panel in the ED.

## Data Availability

Data is available upon request.

## References

[1] Farthing M, Salam MA, Lindberg G, Dite P, Khalif I, Salazar-Lindo E (2013). Acute diarrhea in adults and children: a global perspective. J Clin Gastroenterol.

[2] Lo Vecchio A, Dias JA, Berkley JA, Boey C, Cohen MB, Cruchet S (2016). Comparison of recommendations in clinical practice guidelines for acute gastroenteritis in children. J Pediatr Gastroenterol Nutr.

[3] Myer PA, Mannalithara A, Singh G, Singh G, Pasricha PJ, Ladabaum U (2013). Clinical and economic burden of emergency department visits due to gastrointestinal diseases in the United States. Am J Gastroenterol.

[4] Tate JE, Curns AT, Cortese MM, Weintraub ES, Hambidge S, Zangwill KM (2009). Burden of acute gastroenteritis hospitalizations and emergency department visits in US children that is potentially preventable by rotavirus vaccination: a probe study using the now-withdrawn rotashield vaccine. Pediatrics.

[5] Riddle MS, Connor BA, Beeching NJ, DuPont HL, Hamer DH, Kozarsky P (2017). Guidelines for the prevention and treatment of travelers' diarrhea: a graded expert panel report. J Travel Med..

[6] Kim YJ, Park KH, Park DA, Park J, Bang BW, Lee SS (2019). Guideline for the antibiotic use in acute gastroenteritis. Infect Chemother.

[7] Guerrant RL, Van Gilder T, Steiner TS, Thielman NM, Slutsker L, Tauxe RV (2001). Practice guidelines for the management of infectious diarrhea. Clin Infect Dis.

[8] Yoo IH, Kang HM, Suh W, Cho H, Yoo IY, Jo SJ (2021). Quality improvements in management of children with acute diarrhea using a multiplex-PCR-based gastrointestinal pathogen panel. Diagnostics (Basel)..

[9] Torres-Miranda D, Akselrod H, Karsner R, Secco A, Silva-Cantillo D, Siegel MO (2020). Use of BioFire FilmArray gastrointestinal PCR panel associated with reductions in antibiotic use, time to optimal antibiotics, and length of stay. BMC Gastroenterol.

[10] Truong J, Cointe A, Le Roux E, Bidet P, Michel M, Boize J (2022). Clinical impact of a gastrointestinal PCR panel in children with infectious diarrhoea. Arch Dis Child.

[11] Sever A, Ben Zvi H, Melamed SB, Sachs N, Krause I, Bilavsky E (2023). Clinical impact of biofire gastrointestinal panel testing for hospitalised children with acute gastroenteritis. Acta Paediatr.

[12] Bennett WE, Tarr PI (2009). Enteric infections and diagnostic testing. Curr Opin Gastroenterol.

[13] Hennessy TW, Marcus R, Deneen V, Reddy S, Vugia D, Townes J (2004). Survey of physician diagnostic practices for patients with acute diarrhea: clinical and public health implications. Clin Infect Dis.

[14] Imdad A, Retzer F, Thomas LS, McMillian M, Garman K, Rebeiro PF (2018). Impact of Culture-Independent Diagnostic Testing on Recovery of Enteric Bacterial Infections. Clin Infect Dis.

[15] Buss SN, Leber A, Chapin K, Fey PD, Bankowski MJ, Jones MK (2015). Multicenter evaluation of the BioFire FilmArray gastrointestinal panel for etiologic diagnosis of infectious gastroenteritis. J Clin Microbiol.

[16] Stockmann C, Pavia AT, Graham B, Vaughn M, Crisp R, Poritz MA (2017). Detection of 23 gastrointestinal pathogens among children who present with diarrhea. J Pediatric Infect Dis Soc.

[17] Calderaro A, Martinelli M, Buttrini M, Montecchini S, Covan S, Rossi S (2018). Contribution of the FilmArray((R)) Gastrointestinal Panel in the laboratory diagnosis of gastroenteritis in a cohort of children: a two-year prospective study. Int J Med Microbiol.

[18] Piralla A, Lunghi G, Ardissino G, Girello A, Premoli M, Bava E (2017). FilmArray GI panel performance for the diagnosis of acute gastroenteritis or hemorragic diarrhea. BMC Microbiol.

[19] Xie J, Kim K, Berenger BM, Chui L, Vanderkooi OG, Grisaru S (2023). Comparison of a rapid multiplex gastrointestinal panel with standard laboratory testing in the management of children with hematochezia in a pediatric emergency department: randomized controlled trial. Microbiol Spectr.

[20] Lamberti LM, Fischer Walker CL, Black RE (2012). Systematic review of diarrhea duration and severity in children and adults in low- and middle-income countries. BMC Public Health.

[21] Ory EM, Yow EM (1963). The Use and Abuse of the Broad Spectrum Antibiotics. JAMA.

[22] Colletti JE, Brown KM, Sharieff GQ, Barata IA, Ishimine P, Committee APEM (2010). The management of children with gastroenteritis and dehydration in the emergency department. J Emerg Med.

[23] Carson RA, Mudd SS, Madati PJ (2016). Clinical practice guideline for the treatment of pediatric acute gastroenteritis in the outpatient setting. J Pediatr Health Care.

[24] Guarino A, Ashkenazi S, Gendrel D, Lo Vecchio A, Shamir R, Szajewska H (2014). European Society for Pediatric Gastroenterology, Hepatology, and Nutrition/European Society for Pediatric Infectious Diseases evidence-based guidelines for the management of acute gastroenteritis in children in Europe: update 2014. J Pediatr Gastroenterol Nutr.

[25] Cohen R, Raymond J, Gendrel D (2017). Antimicrobial treatment of diarrhea/acute gastroenteritis in children. Arch Pediatr.

[26] Vukelic D, Trkulja V, Salkovic-Petrisic M (2010). Single oral dose of azithromycin versus 5 days of oral erythromycin or no antibiotic in treatment of campylobacter enterocolitis in children: a prospective randomized assessor-blind study. J Pediatr Gastroenterol Nutr.

[27] Ternhag A, Asikainen T, Giesecke J, Ekdahl K (2007). A meta-analysis on the effects of antibiotic treatment on duration of symptoms caused by infection with Campylobacter species. Clin Infect Dis.

[28] Khan MK, Alam MM (2021). Norovirus gastroenteritis outbreaks, genomic diversity and evolution: an overview. Mymensingh Med J.

[29] Green KY (2014). Norovirus infection in immunocompromised hosts. Clin Microbiol Infect.

[30] Muhlen S, Dersch P (2020). Treatment strategies for infections with shiga toxin-producing *Escherichia coli*. Front Cell Infect Microbiol.

[31] Wong W, Prestidge C, Dickens A, Ronaldson J (2023). Diarrhoea-associated haemolytic uraemic syndrome and Shiga toxin-producing Escherichia coli infections in New Zealand children: Clinical features and short-term complications from a 23-year cohort study. J Paediatr Child Health.

[32] Shane AL, Mody RK, Crump JA, Tarr PI, Steiner TS, Kotloff K (2017). 2017 infectious diseases society of america clinical practice guidelines for the diagnosis and management of infectious diarrhea. Clin Infect Dis.

[33] Geyer B (2020). Diagnosis and management of acute gastroenteritis in the emergency department. Emerg Med Pract.

